# NUAK1 promotes tumor metastasis through upregulating slug transcription in esophageal squamous cell carcinoma

**DOI:** 10.1186/s12935-023-03101-7

**Published:** 2023-11-02

**Authors:** Huiru Yang, Zhen Wei, Yifan Song, Kexin Du, Nannan Yin, Hong Lu, Bingbing Li, Lili Hou, Panfei Xing, Liang Chen, Chaojie Wang, Songqiang Xie

**Affiliations:** 1 School of Pharmacy, Henan University, N. Jinming Ave., Kaifeng, 475004 Henan China; 2 The Academy for Advanced Interdisciplinary Studies, Henan University, N. Jinming Ave., Kaifeng, 475004 Henan China; 3 The Key Laboratory of Natural Medicine and Immuno-Engineering, Henan University, N. Jinming Ave., Kaifeng, 475004 Henan China; 4 Department of Oncology, Huaihe Hospital of Henan University, Kaifeng, 475004 Henan China

**Keywords:** NUAK1, Slug, ESCC, Metastasis, EMT

## Abstract

**Background:**

Metastasis is still a major cause of poor pathological outcome and prognosis in esophageal squamous cell carcinoma (ESCC) patients. NUAK1 has been reported highly expressed in many human cancers and is associated with the poor prognosis of cancer patients. However, the role of NUAK1 and its underlying signaling mechanism in ESCC metastasis remain unclear.

**Methods:**

Expression of NUAK1 in ESCC was detected by real-time quantitative RT-PCR (qRT-PCR), Western blotting and immunohistochemical staining. MTT, colony formation, wound-healing and transwell assays were used to determine the role NUAK1 in vitro. Metastasis was evaluated by use of an experimental pulmonary metastasis model in BALB/c-nu/nu mice. The mechanisms were assessed by using coimmunoprecipitation, immunofluorescence and dual-luciferase reporter gene experiments.

**Results:**

NUAK1 was highly expressed in ESCC tissues compared with the adjacent normal esophageal epithelial tissues. Moreover, the elevated expression of NUAK1 positively correlated with tumor invasion depth, lymph node metastasis, pathological TNM stage, and poor survival in ESCC patients. Further experiments showed that NUAK1 overexpression did not change the cell viability and colony formation of ESCC cells, while remarkably promoted the migration and invasion in vitro and experimental pulmonary metastasis in vivo. Mechanistically, NUAK1 enhanced the transcription level of Slug, which enhanced the migratory and invasive capability of ESCC cells. Consistently, silencing Slug almost completely diminished the migration and invasion of NUAK1-overexpressing ESCC cells. Further studies demonstrated that NUAK1 upregulated the transcription activity of Slug through activating the JNK/c-Jun pathway.

**Conclusion:**

These results demonstrated that NUAK1 promoted the metastasis of ESCC cells through activating JNK/c-Jun/Slug signaling, indicating NUAK1 is a promising therapeutic target for metastatic ESCC.

**Supplementary Information:**

The online version contains supplementary material available at 10.1186/s12935-023-03101-7.

## Background

Esophageal cancer is the seventh most frequently diagnosed cancer and the sixth leading cause of cancer death in the world, with an estimated 604,000 new cases and 544,000 deaths in 2020, according to the GLOBOCAN estimates of cancer incidence and mortality produced by the International Agency for Research on Cancer [1]. Esophageal squamous cell carcinoma (ESCC) is the most common histologic subtype of esophageal cancer in China, accounting for 90% of cases [2]. Although considerable advances achieved in diagnosis and multimodality therapies, the patients with ESCC often have a very poor prognosis, with the 5-year overall survival rate less than 15%, mainly due to the high incidences of tumor metastasis [3]. However, the precise mechanisms underlying the metastasis of ESCC remain unclear. Thus, it is imperative to identify potential molecular biomarkers for the diagnosis and treatment of metastatic ESCC.

Tumor metastasis is a sophisticated cascade process that includes acquisition of invasive and migratory abilities, breach of the basement membrane and detachment from the primary tumor, intrastation, survival in circulation flow, extravasation, adhesion, and colonization at the secondary site [4]. Epithelial mesenchymal transition (EMT), originally be described as an integral part of morphogenesis in embryonic development, has been shown to be a critical first step for the metastatic cascade process [5]. EMT is characterized by the loss of adherence junctions and apical-basal polarity, acquisition of mesenchymal phenotype, and the gain of migratory and invasive traits [6]. Undergoing EMT, cancer cells usually exhibit both morphological changes and molecular alterations, as demonstrated by lost expression of epithelial markers such as E-cadherin, ZO-1, and occludin, and increased expression of mesenchymal markers, including N-cadherin, vimentin, fibroblast-specific protein 1, and fibronectin [5]. In addition, EMT transcription factors (Snail, Slug, Zeb and Twist, etc.) are activated to orchestrate the EMT program [7]. However, mechanistic understanding of EMT in metastasis is still limited.

Novel (nua) kinase family 1 (NUAK1), also known as AMP-activated protein kinase (AMPK)-related protein kinase 5, is a serine/threonine kinase that has been identified as the fifth member of the AMPK catalytic subunit family [8]. Aberrant expression of NUAK1 has been observed in patients diagnosed with multiple cancer types including prostate cancer [9], nasopharyngeal carcinoma [10], pancreatic cancer [11] and colorectal cancer [12]. The high NUAK1 expression positively correlated with tumor stage, invasiveness, poor differentiation, lymph node metastasis [13] and the reduced overall survival of cancer patients [11]. The pro-metastatic function of NUAK1 was first uncovered in pancreatic cancer and colon cancer, which was supported by the observation that ectopic overexpression of NUAK1 in human pancreatic cancer cell line PANC-1 and human colon cancer cell line DLD-1 induced a dramatic increase in migration and invasion activity in vitro and metastasis in vivo [14]. Subsequently, the pro-metastatic function of NUAK1 has also been demonstrated in breast cancer [15], head and neck cancer [16] and hepatocellular carcinoma [17]. These findings indicated that NUAK1 is a potential target for metastatic malignancies. Indeed, knockdown of NUAK1 remarkably suppressed the tumor cell invasion and metastasis in gastric cancer [18], nasopharyngeal carcinoma [19] and ovarian cancer [20]. However, the expression and potential role of NUAK1 in ESCC has never been investigated.

In this study, both gain- and loss-of-function studies showed that NUAK1 did not influence the cellular proliferation and colony formation, while remarkably promoted the migration, invasion and metastasis in ESCC. We further demonstrated that NUAK1 promoted the transcription activity of Slug through activating the JNK/c-Jun pathway. Collectively, our findings highlight the oncogenic role of NUAK1 in promoting ESCC cell migration, invasion and metastasis through activating JNK/c-Jun/Slug signaling, implicating that NUAK1 is a potential therapeutic target for the metastatic ESCC.

## Materials and methods

### Clinical samples

All ESCC tissues and the paired adjacent normal tissues used in this study were obtained from January 2016 to November 2022 in the Huaihe Hospital of Henan University. All the patients enrolled in the research haven’t received radiation or chemotherapy treatment before surgery. All processes were approved by the Ethics Committees of Henan University (Kaifeng, China) and written informed consent was obtained from each patient prior to sample collection according to the principles of Helsinki Declaration. The immunohistochemistry (IHC) staining was performed as described previously [21]. The tumor tissues were stained with anti-NUAK1 antibody (1:100) and scored. The staining intensity was classified into four degrees (0, no staining; 1, low intensity; 2, moderate intensity; and 3, strong intensity). The final IHC staining scores = (score of the staining intensity) × (the proportion of positively stained cells) [21].

### Cell lines and cell culture

The human ESCC cell lines EC9706, EC109, KYSE70, KYSE510, KYSE150, and KYSE30, and one normal esophageal epithelial cell line (Het-1A) were cultured as our previously described [22]. All cells were maintained in a humidified atmosphere (5% CO_2_) at 37 °C and were recently tested for STR profiling and mycoplasma contamination.

### Antibodies and reagents

The anti-NUAK1 antibody (22723-1-AP) and anti-β-actin antibody (23660-1-AP) were purchased from ProteinTech (Chicago, IL, USA); the anti-E-cadherin (#14472), anti-Vimentin (#46173), anti-N-cadherin antibody (#13116), TWIST1 (#90445), ZEB1 (#83243), Snail (#3879), Slug (#9585), p-c-Jun (S73) (#3270), c-Jun (#9165), p-JNK (T183/Y185) (#4668), JNK (#9252), p-Erk1/2 (T202/Y204) (#4370), Erk1/2 (#4695), p-p38 (T180/Y182) (#54470) and p38 (#9212) were purchased from Cell Signaling Technology (CST, Danvers, MA, USA). SP600125 (HY-12,041), JNK-IN-8 (HY-13,319) and puromycin dihydrochloride (HY-B1743A) were purchased from MCE (NJ, USA).

### Quantitative real-time polymerase chain reaction (qRT-PCR)

For this experiment, RNA extraction was performed as previously described [21]. Briefly, total RNA was extracted from cultured cell lines or frozen clinical tissues using Trizol reagent (Invitrogen, USA), and 1 µg of the total RNA was reverse transcribed with a Revert Aid First Strand cDNA Synthesis Kit (#K1622, Thermo, USA) according to the manufacturer’s protocol. qRT-PCR was performed with LightCycler 480 SYBR Green I Master (Roche, Basel, Switzerland) using the LightCycler® 480 Instrument II (Roche Diagnostics AG, Rotkreuz, Switzerland). The sequences of the primers used for amplifying NUAK1, Slug and GAPDH are listed in Additional file 1: Table S1. Relative mRNA expression was calculated using the 2^−ΔΔCt^ method and normalized to those of the internal control gene GAPDH.

### Western blot analysis

Total protein was extracted from cultured cells and analyzed as previously described [22]. Briefly, the whole cell lysates were prepared in RIPA buffer containing 1× protease inhibitor cocktail (Roche, Indianapolis, IN), 1 mM phenylmethylsulfonyl fluoride, 10 mM β-glycerophosphate and 10 mM NaF. The concentration of total protein was quantified by using Pierce™ BCA Protein Assay Kit (#23250, Thermo, USA). A total of 30 µg of protein extracts were loaded to 10% sodium dodecyl sulfate-polyacrylamide gel electrophoresis (SDS-PAGE) and transferred to PVDF membranes (Millipore, USA). After blocking with 5% dried skimmed milk for 1 h at room temperature, the membrane was incubated with the primary antibodies overnight at 4 °C and then with HRP conjugated secondary antibodies (Promega, USA) for 1 h at room temperature, Protein bands were detected using enhanced chemiluminescence (ECL) detection reagent (Beyotime, Shanghai, China) on a FluorChem M system (Protein Simple, USA).

### Co-immunoprecipitation (Co-IP) assay

This experiment was performed using the Immunoprecipitation Kit with Protein A Magnetic Beads (#P2175S, Beyotime, Shanghai, China) according to the manufacturer’s instructions. Briefly, cells were harvested in lysis buffer containing protease inhibitor cocktail and phosphatase inhibitor cocktail A (#P1081, Beyotime, Shanghai, China), incubated for 30 min on ice and centrifuged at 12,000×*g* at 4 °C for 10 min. Cell lysates were immunoprecipitated with antibodies against NUAK1 and JNK or negative control IgG and protein A magnetic bead overnight with rotation. After being washed three times with lysis buffer, these beads were resuspended in 50 µL SDS-PAGE sample loading buffer, denaturalized at 100 °C for 5 min and subjected to Western blot analysis.

### Plasmid construction and lentivirus Infection

The constructs encoding full-length human NUAK1, Slug and c-Jun cDNA were subcloned into the pLVX-IRES-neo vector. The pLVX-IRES-neo empty vector was used as negative control. Non-targeting (mock) siRNA (#sc-37007) and siRNA oligoduplexes against c-Jun (#sc-29223) were obtained from Santa Cruz Biotechnology (Santa Cruz, CA, USA). Scramble (pLKO.1-puro-Non-target shRNA), specific target shRNAs against human NUAK1 or Slug were purchased from Sigma-Aldrich (Shanghai, China). Cells were transfected with siRNA duplexes using Lipofectamine™ RNAiMAX Transfection Reagent (#13778100, Thermo, USA) according to the manufacturer’s instructions. The transfection of overexpressing plasmids and shRNA constructs was carried out using Lipofectamine™ 3000 Transfection Reagent (#L3000015, Thermo, USA). For lentivirus production, the indicated lenti-vectors and the packaging plasmids (pCMV-dR8.2 dvpr and pCMV-VSV-G) were cotransfected into 293T cells. The culture medium containing mature lentivirus particles was collected and purified with 0.45-µm filters at 48 h after transfection. Then the indicated lentivirus and polybrene (8 µg/mL) were added to ESCC cells, after infection for 24 h, cells were selected with puromycin (1.5 µg/mL) or G418 (800 µg/mL) for 2 weeks to establish stable cell populations.

### MTT assay

The MTT assay was conducted to examine the effects of NUAK1 overexpression and knockdown on the viability of ESCC cells as described previously [22]. Briefly, cells were seeding into 96-well culture plates at the density of 1000 cells/well and assessed at five time points (on days 1, 2, 3, 4, and 5). Twenty microliters of MTT (Sigma-Aldrich, USA) reagent (5 mg/mL) was added into each well, and the plates were incubated at 37 °C for another 4 h. After discarding the supernatant, one hundred microliters of DMSO was added to dissolve the formed formazan crystals. Then the optical density (OD) value was measured by a spectrophotometric plate reader (Synergy HT; BioTek, Winooski, VT, USA) at 570 nm. Each group was comprised of four duplicated wells, and the assay was performed five times independently.

### Foci formation and soft agar assays

For foci formation assay, cells (500/well) stably transfected with NUAK1 or NUAK1 shRNA were seeded into 24-well plates and cultured for 14 days. Colonies composing containing ≥ 50 cells were fixed and stained with 0.1% crystal violet in 20% methanol for 20 min and counted using an inverted microscope (Olympus, Japan). For soft agar assay, cells (2000/well) mixed with 0.4% Bacto-agar (#A5306, Sigma-Aldrich, Shanghai, China) were plated on a bottom layer of solidified 0.8% agar in a 24-well plate. Two weeks later, the surviving colonies composing more than 50 cells were determined by microscope counting.

### Immunofluorescence

Briefly, cells were washed with PBS, fixed with 4% paraformaldehyde for 20 min, permeabilized with 0.5% Triton X-100 for 15 min and blocked with 3% bovine serum albumin in PBS for 1 h at room temperature. Cells were then incubated overnight with primary antibodies: E-cadherin (#14472, 1:200, CST) and Vimentin (#46173, 1:100, CST), After washing with PBS, cells were incubated with anti-mouse Alexa Fluor 594 antibody (#8890, 1:1000, CST) or anti-rabbit Alexa Fluor 488 antibody (#4412, 1:1000, CST) at room temperature. After washing thrice with PBS, the cells were by incubation for 5 min with DAPI (#4083, 0.5 µg/mL, CST) to label the nuclei. Images were captured using a confocal scanning microscope (Leica TCS SP8, Germany).

### Wound-healing assay

Cells were seeded in 6-well plates at a density of 5 × 10^5^ cells/well and cultured until 80–90% confluence. A 10 µL sterile pipette tip was used to introduce a straight scratch simulating a wound, the wells were washed with PBS to remove the floating cells, and the medium was then replaced with serum-free culture medium. Images were captured at 0 h, 24 and 48 h after scratching using an inverted microscope.

### Cell migration assay and invasion assays

This experiment was performed using chambers with polyethylene terephthalate membranes (8 μm pore size) (BD Biosciences, USA) as previously described [22]. Briefly, cells were suspended in serum-free RPMI 1640 medium and added to the upper chamber, which was coated without (migration assay) or with (invasion assay) the Matrigel mix (BD Biosciences, USA). Culture medium supplemented with 10% FBS was placed into the lower chamber as a chemo-attractant. After 24 h of incubation, the migrated or invaded cells on the bottom of the membrane were stained with 0.1% Crystal Violet. The total number of cells was counted from six random fields using an inverted microscope (Olympus, Japan). All experiments were performed in duplicate and repeated five times.

### Dual-luciferase reporter gene assay

Cells were plated in 24-well plates and cultured for overnight. The cells were then cotransfected with the appropriate pGL3-Slug promoter-luciferase plasmid or pGL3-luciferase control plasmid, together with the renilla luciferase control reporter using the Lipofectamine 3000 kit (#L3000015, Thermo, USA). Forty-eight hours later, the luciferase activities in the cell lysates were measured using the Dual-Luciferase® Reporter Assay System (#E1910, Promega, USA) in accordance with the manufacturer’s instructions and normalized with renilla luciferase activity. All of the experiments were performed in five times.

### In vivo tumor metastasis

All animal experiments were approved by the Henan University Institutional Animal Care and Use Committee. Five-week-old male BALB/c-nu/nu mice purchased from Vital River (Beijing, China) were housed in specific pathogen-free (SPF) conditions in a laboratory animal facility with controlled temperature (20 ± 2 °C), humidity (40–50%) and a lighting cycle of 12 h darkness/12 h light.

For the in vivo metastasis assays, 2 × 10^6^ NUAK1-overexpressing EC109 cells or NUAK1-knockdown KYSE30 cells and control cells were separately injected into the lateral tail veins of nude mice (6 per group). After 8 weeks, the mice were sacrificed with isoflurane, and their lungs were harvested at necropsy and fixed in Bouin’s solution. The numbers of metastatic colonies on the lung surface of each mouse were carefully counted, and histological evidence of the lung tissues was further examined by hematoxylin and eosin (H&E) or IHC staining.

### Database analysis

the mRNA level of NUAK1 in TCGA ESCC samples was analyzed using UALCAN database (http://ualcan.path.uab.edu/index.html). The NUAK1 transcriptome data downloaded from Gene Expression Omnibus (GEO) database (Accession Number GSE23400, GSE44021 and GSE53625) (https://www.ncbi.nlm.nih.gov/geo/) was analyzed using the limma packages for GEO data by R software.

### Statistical analysis

The data are presented as the mean ± the standard deviation (SD) of at least three independent experiments. The statistical analysis was performed using SPSS software version 22.0 (Chicago, IL, USA) and all graphs were performed by GraphPad Prism software 7.0 (San Diego, CA, USA). Student’s t-test or paired t-test was used to compare the differences between two groups. We compared 3 or more groups with one-way ANOVA with Tukey’s post hoc test, the overall F test was significant (P < 0.05), and there was no significant variance in homogeneity. Survival curves were constructed by the Kaplan–Meier method, and a log-rank test was used for comparison. All statistical tests were two-sided, and a *P* value of less than 0.05 was regarded as statistically significant.

## Results

### NUAK1 is highly elevated in ESCC

**Fig. 1:**
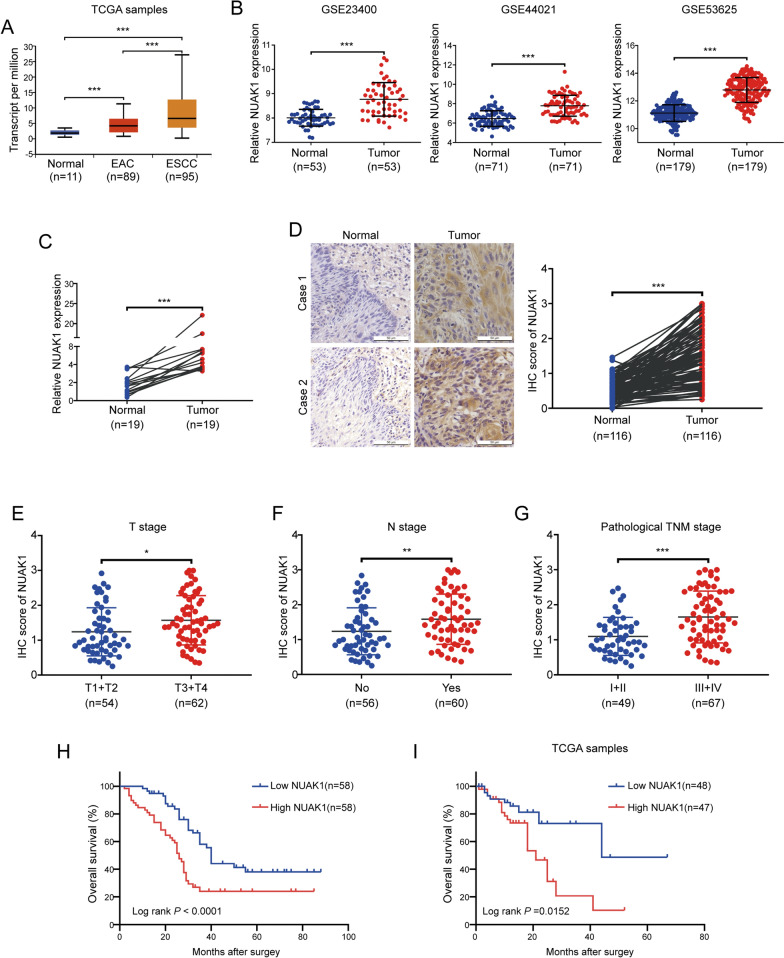
NUAK1 is highly expressed in ESCC.** A** The mRNA level of NUAK1 was analyzed in normal and cancerous tissues from the TCGA cohort. **B** Data from the GEO database showed that NUAK1 was upregulated in ESCC tissues compared to the adjacent normal counterparts. **C** qRT-PCR analysis of the levels of NUAK1 mRNA in 19 pairs of ESCC tumor and matched normal tissues. **D** IHC staining for NUAK1 was performed in 116 pairs of ESCC tissues and adjacent normal tissues. Representative images (left panel) and IHC scores (right panel) are shown (scale bar, 100 μm). *P* value was calculated by paired t-test (for **A**–**D**). **E**–**G** The NUAK1 staining score for T stage (**E**), N stage (**E**) and pathological TNM stage (**E**) in ESCC. *P* value was calculated by Student’s t-test (for **E**–**G**). **H** Kaplan–Meier analysis revealed that high NUAK1 expression predicted poor prognosis in ESCC. **I** Survival analysis of TCGA patients from whom the ESCC samples were obtained based on the NUAK1 expression level. *P* value was calculated by the log-rank test (for **H** and **I**). Error bars denote mean ± SD. *P < 0.05; **P < 0.01; ***P < 0.001

To determine the expression of NUAK1 in ESCC, we first analyzed the mRNA level of NUAK1 using TCGA samples from UALCAN database (http://ualcan.path.uab.edu/index.html), the results showed that NUAK1 was highly expressed in ESCC specimens (n = 95) compared to that in esophageal adenocarcinoma (EAC) (n = 89) and the adjacent normal tissues (n = 11) (Fig. 1A). We further analyzed the expression of NUAK1 with three GEO datasets (GSE23400, GSE44021 and GSE53625). As shown in Fig. 1B, the mRNA level of NUAK1 was much higher in ESCC tissues compared with the adjacent normal counterparts. To verify the bioinformatic analysis results, we examined a set of ESCC and paired adjacent normal tissues, confirming that NUAK1 mRNA levels were upregulated in cancerous tissues, compared to the paired adjacent normal counterparts (Fig. 1C). To explore the protein expression and localization of NUAK1 in ESCC tissues, we performed IHC staining on ESCC sections. As illustrated Fig. 1D, NUAK1 was highly expressed in ESCC tissues compared with that in adjacent esophageal epithelial tissues and it is mainly expressed in the cytoplasm and nucleus of ESCC cells. Further analysis showed that the elevated NUAK1 expression was positively correlated with T stage (primary tumor invasion depth), N stage (lymph node metastasis) and pathological TNM stage but has no correlation with age, gender, tumor location or differentiation (Fig. 1E–G and Additional file 1: Table S2). Kaplan–Meier analysis revealed that high NUAK1 expression in ESCC patients was positively associated with reduced overall survival (Fig. 1H). Consistently, the positive correlation between NUAK1 expression and poor prognosis was further confirmed in TCGA ESCC samples (Fig. 1I). Taken together, these results indicate that NUAK1 is aberrantly expressed in ESCC tissues and probably play an oncogenic role in ESCC progression and metastasis.

### NUAK1 does not affect the growth of ESCC cells

**Fig. 2:**
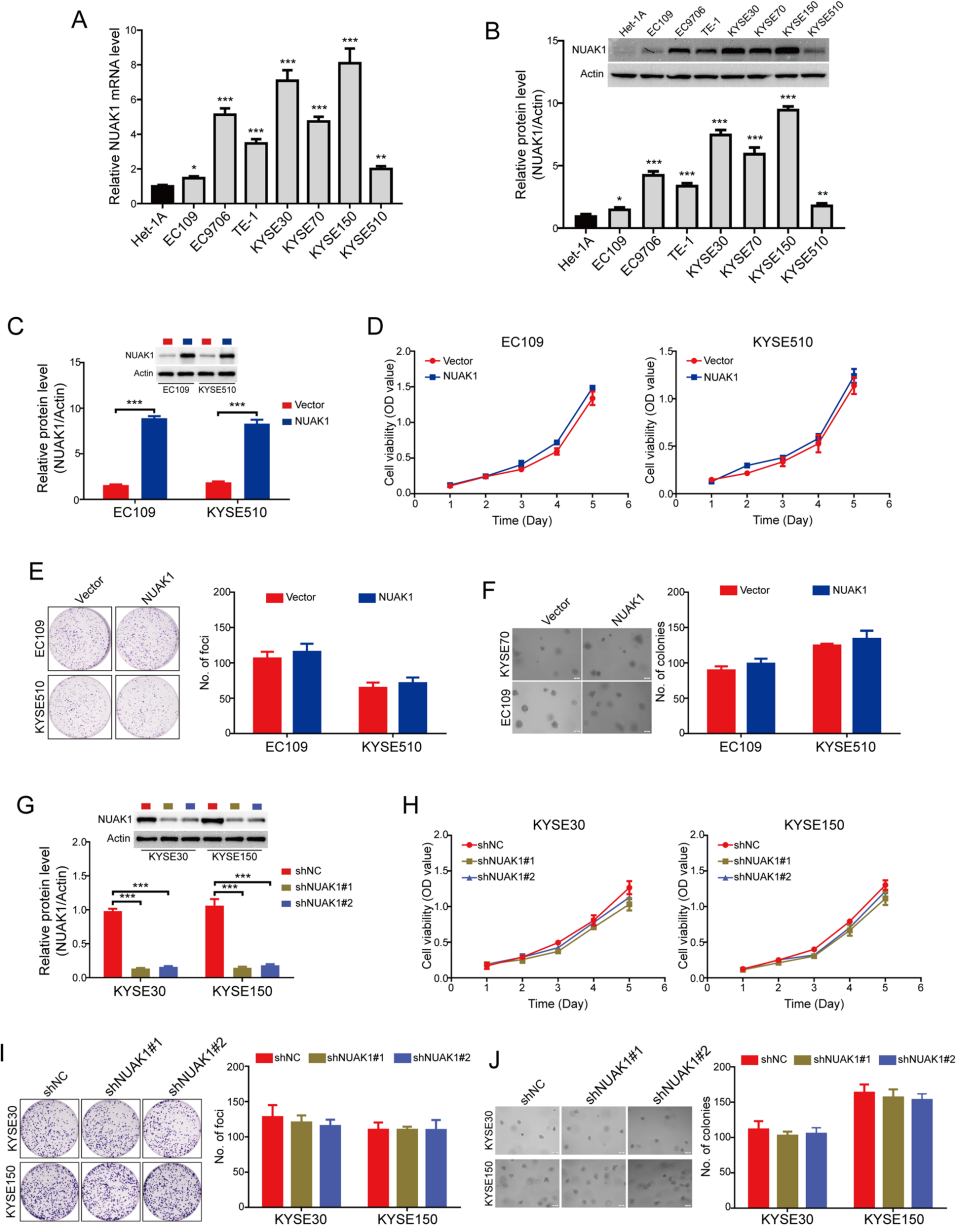
NUAK1 does not influence the tumor cell growth in ESCC cells.** A**, **B** The expression of NUAK1 in ESCC cells at mRNA and protein levels were detected by qRT-PCR (**A**) and Western blotting (**B**), respectively. *P* value was calculated by one-way ANOVA with post hoc intergroup comparison by the Tukey’s test. **C** Western blot analysis was performed to detect the level of NUAK1 in EC109 and KYSE510 cells stably expressing NUAK1. Quantification of indicated protein levels of n = 3 independent biological experiments (low panel). **D**–**F** The effects of NUAK1 overexpression on ESCC cell growth were assessed by MTT assay (**D**), foci formation assay (**E**) and soft agar assay (**F**). *P* value was calculated by Student’s t-test (for **C**–**F**). **G** KYSE30 and KYSE150 cells stably expressing shNC or NUAK1 shRNA as indicated were analyzed by Western blotting. Quantification of indicated protein levels of n = 3 independent biological experiments (low panel). **H**–**J** The effects of NUAK1 knockdown on ESCC cell growth were assessed by MTT assay (**H**), foci formation assay (**I**) and soft agar assay (**J**). *P* value was calculated by one-way ANOVA with post hoc intergroup comparison by the Tukey’s test (for **G**–**J**). Error bars denote mean ± SD. *P < 0.05; **P < 0.01; ***P < 0.001

To further investigate the potential role of NUAK1 in regulating the malignant process of ESCC, we first detected the mRNA and protein levels of NUAK1 in seven ESCC cell lines (EC109, EC9706, TE-1, KYSE30, KYSE70, KYSE150 and KYSE510) and an immortalized esophageal epithelial cell line (Het-1 A). The results showed that elevated NUAK1 expression was observed in all tested ESCC cell lines, compared with the immortalized Het-1 A esophageal epithelial cell line (Fig. 2A, B). We used lentiviruses to overexpress NUAK1 in EC109 and KYSE510 cells, which endogenously express a lower level of NUAK1 among the tested ESCC cells. The transfection efficiency of NUAK1 was confirmed by Western blot analysis (Fig. 2C). MTT cell growth assay showed that ectopic expression of NUAK1 did not affect the cell viabilities of EC109 and KYSE510 cells (Fig. 2D). We further examined whether overexpression of NUAK1 promotes the colony formation using foci formation assay and soft agar assay. However, no significant differences were observed between NUAK1-overexpressing and control cells (Fig. 2D, E). Next, we determined whether knocking down NUAK1 suppresses the tumorigenic ability of ESCC cells. Two pairs of small hairpin RNAs (shRNAs) against NUAK1 were used to dramatically reduce endogenous NUAK1 expression in KYSE30 and KYSE150 cells, which endogenously express a high level of NUAK1 (Fig. 2G). The results revealed that the cell viability and colony formation of KYSE30 and KYSE150 cells was just marginally inhibited by NUAK1 shRNA, compared with the control group (Fig. 2H–J). Collectively, these data together suggest that NAUK1 does not influence the growth of ESCC cells.

### NUAK1 promotes ESCC cell migration and invasion

**Fig. 3:**
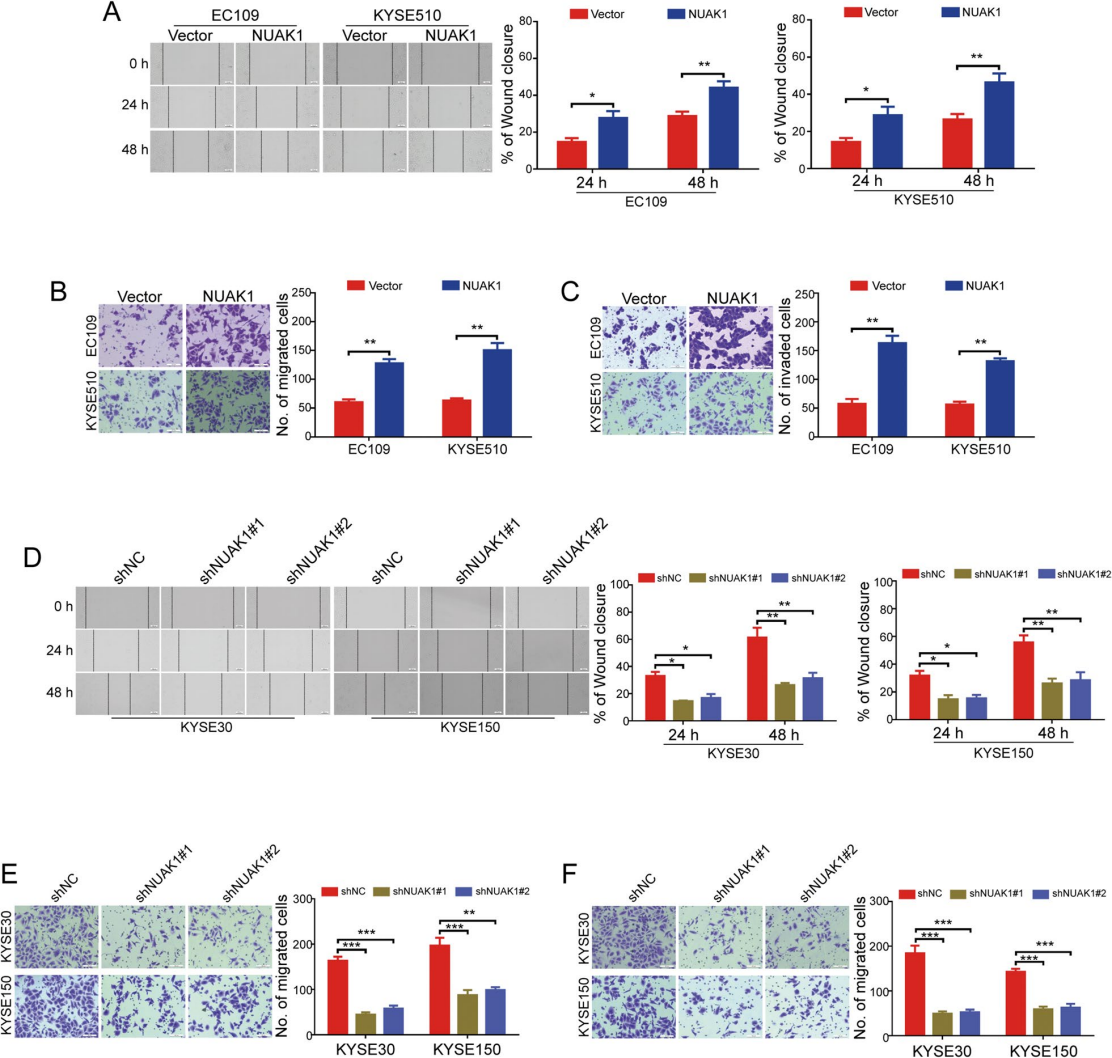
NUAK1 promotes the migration and invasion of ESCC cells.** A**–**C** The effects of NUAK1 overexpression on ESCC cell migration and invasion were assessed by wound healing assay (**A**), transwell migration (**B**), and invasion assay (**C**). *P* value was calculated by Student’s t-test. **D**–**F** The effects of NUAK1 knockdown on ESCC cell migration and invasion were assessed by wound healing assay (**D**), transwell migration (**E**), and invasion assay (**F**). *P* value was calculated by one-way ANOVA with post hoc intergroup comparison by the Tukey’s test. Error bars denote mean ± SD. *P < 0.05; **P < 0.01; ***P < 0.001

Subsequently, we sought to determine the potential role of NUAK1 on the migration and invasion of ESCC cells. Wound-healing scratch assay showed that ectopic overexpression of NUAK1 greatly enhanced the wound-closure capability of EC109 and KYSE510 cells (Fig. 3A). Similarly, the NUAK1-overexpressing ESCC cells showed a significantly increased migration ability using transwell migration assay (Fig. 3B). Assessed by transwell invasion assays, overexpression of NUAK1 dramatically increased the number of invaded ESCC cells (Fig. 3C). Considering overexpression of NUAK1 promoted the migration and invasion of ESCC cells, we then sought to examine whether silencing NUAK1 attenuates these malignant behaviors. As shown in Fig. 3D, knockdown of NUAK1 significantly abolished the motility of KYSE30 and KYSE150 cells. Consistent with this result, transwell migration assay also showed that the NUAK1-knockdown ESCC cells showed a significantly decreased migration ability, compared with those cells with shNC transfection (Fig. 3E). In addition, the number of tumor cells invaded through Matrigel were decreased in both tested NUAK1-depleted ESCC cells (Fig. 3F). Collectively, these data demonstrate that NUAK1 overexpression promotes the migratory and invasive capability of ESCC cells.

### NUAK1 promotes slug-mediated EMT in ESCC cells

**Fig. 4:**
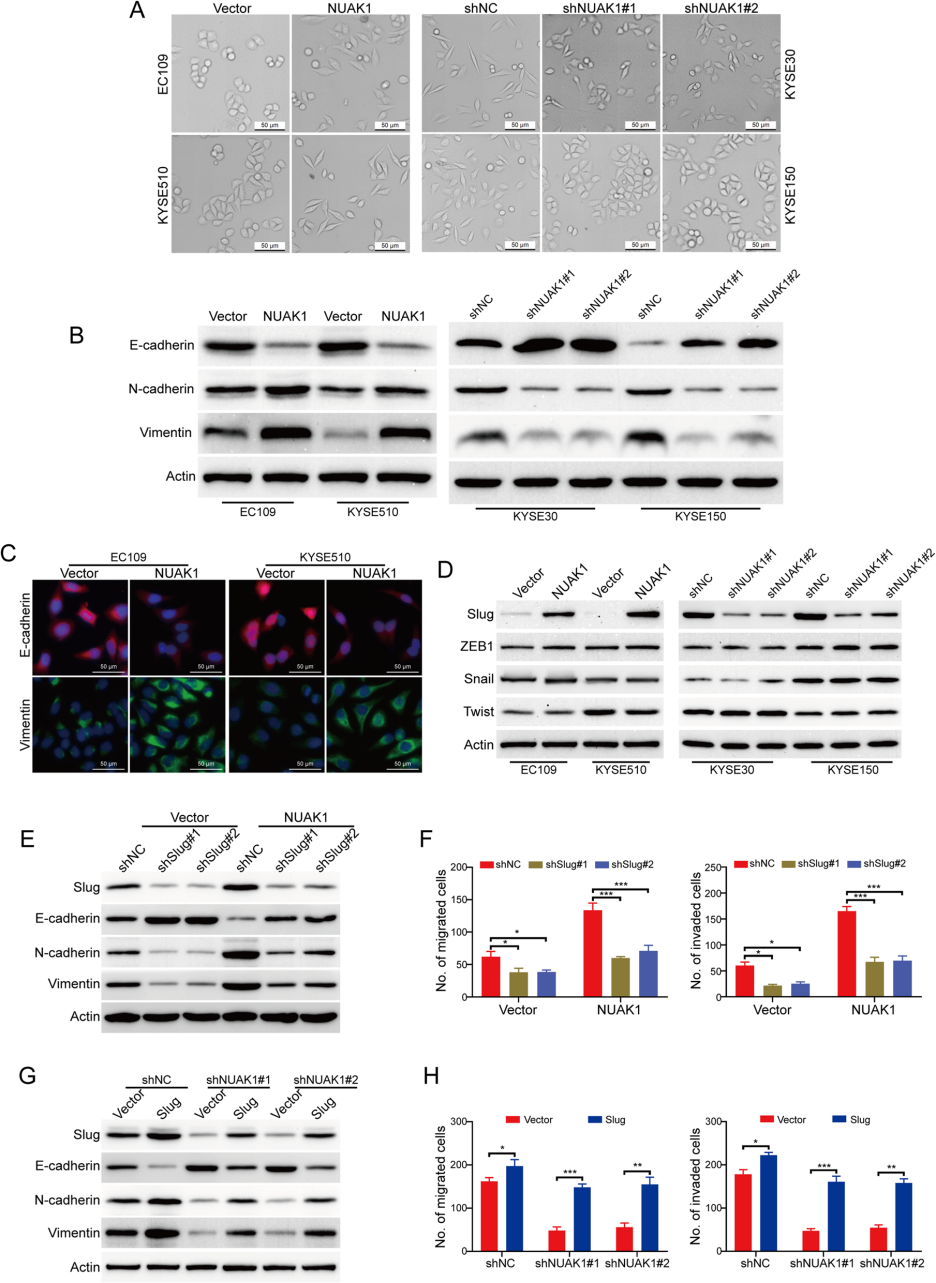
NUAK1 promotes ESCC cell migration and invasion via Slug-mediated EMT.** A** The effects of NUAK1 overexpression and knockdown on the morphological changes of ESCC cells were shown. **B** The effects of NUAK1 overexpression and knockdown on the protein levels of E-cadherin, N-cadherin and Vimentin were measured by Western blotting. **C** The effects of NUAK1 overexpression on E-cadherin and Vimentin were analyzed by immunofluorescence assay. **D** Relative expressions of the indicated molecules were detected by Western blotting. **E** Western blot analyses of the levels of Slug, E-cadherin, N-cadherin and Vimentin in NUAK1-overexpressing EC109 cells treated with Slug shRNA. **F** Transwell assay was used to detect the migration (left panel) and invasion (right panel) in NUAK1-overexpressing EC109 cells treated with Slug shRNA. **G** Western blot analyses of the levels of Slug, E-cadherin, N-cadherin and Vimentin in NUAK1-knockdown KYSE30 cells treated with Slug and control plasmids. **H** Transwell assay was used to detect the migration (left panel) and invasion (right panel) in NUAK1-knockdown KYSE30 cells treated with Slug and control plasmids. *P* value was calculated by one-way ANOVA with post hoc intergroup comparison by the Tukey’s test. Error bars denote mean ± SD. *P < 0.05; **P < 0.01; ***P < 0.001

Increasing reports have shown that EMT is associated with cancer migration, invasion and metastasis [5, 23, 24]. Because NUAK1-overexpressing cells exhibited a more fibroblast-like mesenchymal features compared with their control cells, which displayed epithelial-like phenotype (Fig. 4A). In addition, we also noticed that the morphology of stable NUAK1-knockdown cells has been changed from the spindle-like and fibroblastic phenotype (mesenchymal feature) into the tight cell-to-cell adhesion (epithelial-like phenotype) (Fig. 4A), suggesting NUAK1 promoted EMT in ESCC cells. We then decided to detect whether NUAK1 influence the EMT-related proteins in NUAK1-overexpressing and knockdown cells using Western blot analysis. As shown in Fig. 4B and Additional file 1: Fig. S1A, we found that ectopic overexpression of NUAK1 inhibited the expression of E-cadherin, while enhanced the expression of the mesenchymal markers, N-cadherin and Vimentin. In contrast, silencing NUAK1 inhibited the expression of the mesenchymal markers, N-cadherin and Vimentin, while reducing E-cadherin expression in both tested NUAK1-knockdown cells (Fig. 4B and Additional file 1: Fig. S1A). Moreover, immunofluorescence assay also showed that ectopic expression of NUAK1 downregulated E-cadherin expression, while upregulating Vimentin expression in EC109 and KYSE510 cells (Fig. 4C). Considering the crucial role of EMT-related transcription factors (Twist, ZEB1, Snail and Slug) for EMT, we then detected whether NUAK1 altered their expression using immunoblotting analysis. Strikingly, only Slug was remarkably increased in NUAK1-overexpressing cells and was diminished in NUAK1-knockdown cells (Fig. 4D and Additional file 1: Fig. S1B). To further investigate the role of Slug in NUAK1-mediated EMT, we used two pairs of small hairpin RNAs (shRNAs) to dramatically reduce endogenous Slug expression in NUAK1-overexpressing EC109 cells (Fig. 4E and Additional file 1: Fig. S1C). As expected, silencing Slug remarkably rescued the expression of E-cadherin, while diminishing the levels of N-cadherin and Vimentin induced by NUAK1 overexpression (Fig. 4F and Additional file 1: Fig. S1D). On the other hand, Slug was transiently transfected into the NUAK1-silenced KYSE30 cells, and the migratory and invasive abilities were almost rescued to the original levels in the KYSE30 cells (Fig. 4G, H). Taken together, our results indicate that NUAK1 promotes migration and invasion via Slug-mediated EMT in ESCC cells.

### c-Jun is crucial for slug transcription induced by NUAK1 in ESCC cells

**Fig. 5:**
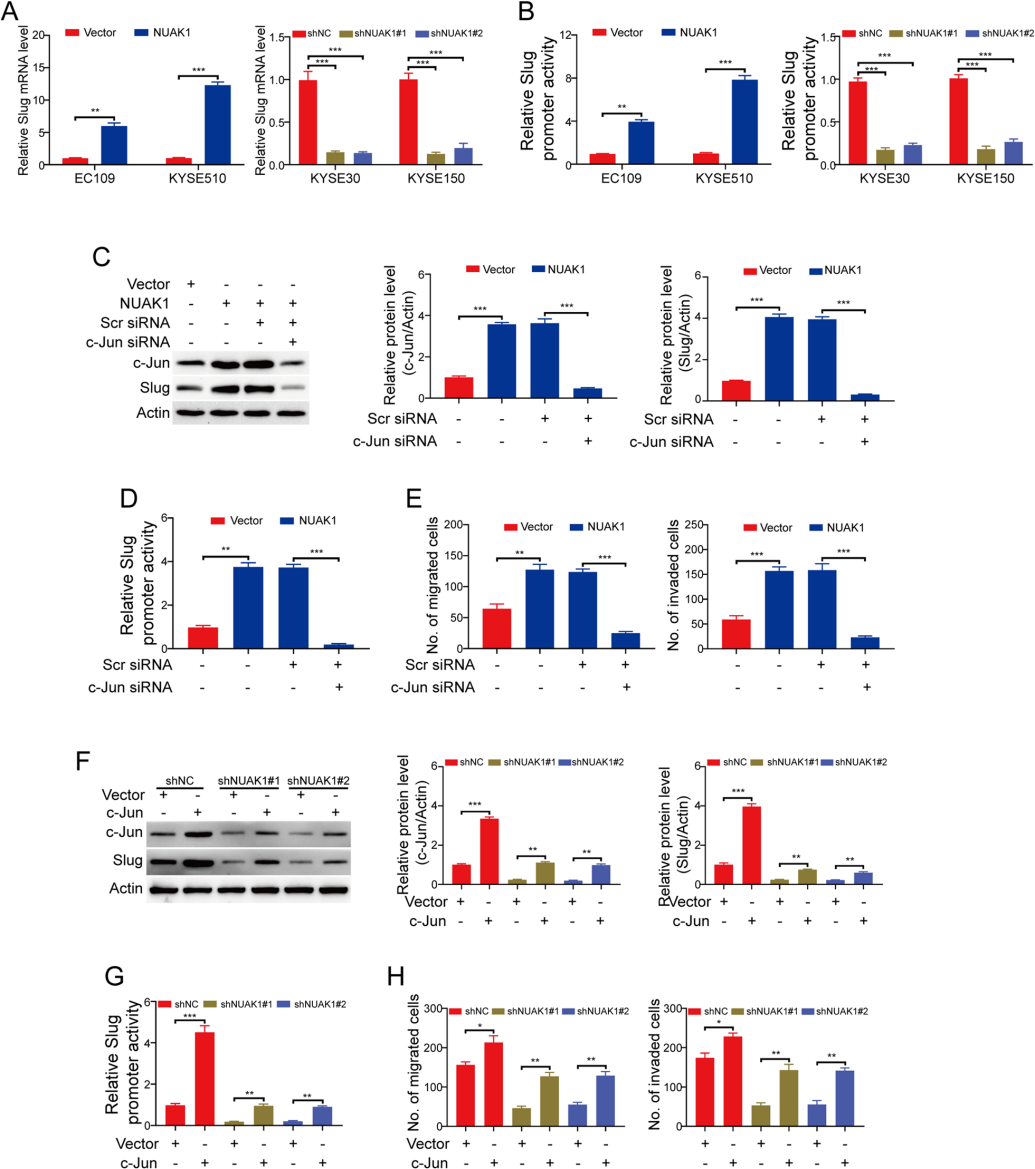
c-Jun is crucial for NUAK1-induced migration and invasion in ESCC cells.** A** The expression of Slug mRNA was detected using qRT-PCR analysis. **B** The promoter activity of Slug was analyzed using the luciferase assay in NUAK1-overexpressing or knockdown ESCC cells. **C** The expression of c-Jun and Slug was analyzed in NUAK1-overexpressing EC109 cells treated with c-Jun shRNA. Quantification of indicated protein levels of n = 3 independent biological experiments (right panel). **D** The promoter activity of Slug was analyzed using the luciferase assay in NUAK1-overexpressing EC109 cells treated with c-Jun shRNA. **E** Cell migration (left panel) and invasion (right panel) were analyzed in NUAK1-overexpressing EC109 cells treated with c-Jun shRNA. **F** The expression of c-Jun and Slug was analyzed in NUAK1-knockdown KYSE30 cells treated with c-Jun and control vector plasmids. Quantification of indicated protein levels of n = 3 independent biological experiments (right panel). **G** The promoter activity of Slug was analyzed using the luciferase assay in NUAK1-knockdown KYSE30 cells treated with c-Jun and control vector plasmids. **H** Cell migration (left panel) and invasion (right panel) were analyzed in NUAK1-knockdown KYSE30 cells treated with c-Jun and control plasmids. Error bars denote mean ± SD. *P < 0.05; **P < 0.01; ***P < 0.001. *P* value was calculated by Student’s t-test (for **A** and **B**, left panel) or one-way ANOVA with post hoc intergroup comparison by the Tukey’s test (for **A** and **B**, right panel; **D**, **E**, **G** and **H**)

To explore how NUAK1 promotes Slug expression, the experiment of qRT-PCR was carried out to examine the mRNA level of Slug in ESCC cells. The results showed that the transcription of Slug was upregulated in NUAK1-overexpressing EC109 and KYSE510 cells, while was downregulated in NUAK1-depleted KYSE30 and KYSE150 cells (Fig. 5A). Considering that c-Jun has been identified as a critical transcription factor to regulate Slug expression through the AP1 site in the Slug promoter [25]. To determine whether c-Jun participates in Slug transcription induced by NUAK1 in ESCC cells, we constructed a slug promoter reporter plasmid by linking the slug promoter to a luciferase gene and transfected this plasmid into the tested ESCC cells. Assaying luciferase activity showed that slug promoter activity was significantly enhanced by NUAK1 in NUAK1-overexpressing EC109 and KYSE510 cells, while was inhibited by NUAK1 shRNA in NUAK1-depleted KYSE30 and KYSE150 cells (Fig. 5B). Moreover, silencing c-Jun by siRNA duplexes against c-Jun drastically diminished the expression of Slug in NUAK1-overexpressing EC109 cells (Fig. 5C). Consistently, luciferase reporter assay also showed that knocking down c-Jun greatly abrogated the Slug promoter activity induced by NUAK1 (Fig. 5D). These results suggest that c-Jun is crucial for NUAK1-induced Slug transcription in ESCC. To further determine whether c-Jun is involved in the migration and invasion induced by NUAK1, we performed transwell migration and invasion assay. As shown in Fig. 5E, silencing c-Jun remarkably reduced the numbers of migrated and invaded cells induced by NUAK1. Subsequently, we examined whether overexpression of c-Jun attenuates the inhibitory effects of NUAK1 shRNA on Slug expression. The results showed that forced overexpression of c-Jun drastically rescued the expression and promoter activity of Slug mediated by NUAK1 shRNA (Fig. 5F, G). As expected, the migration and invasion of NUAK1-depleted KYSE30 cells were obviously enhanced by c-Jun overexpression (Fig. 5H). These findings together indicate that NUAK1 promotes the transcription of Slug induced by c-Jun, and thus enhances ESCC cell migration and invasion.

### NUAK1 activates the JNK/c-Jun pathway to derive slug-mediated migration and invasion in ESCC cells

**Fig. 6:**
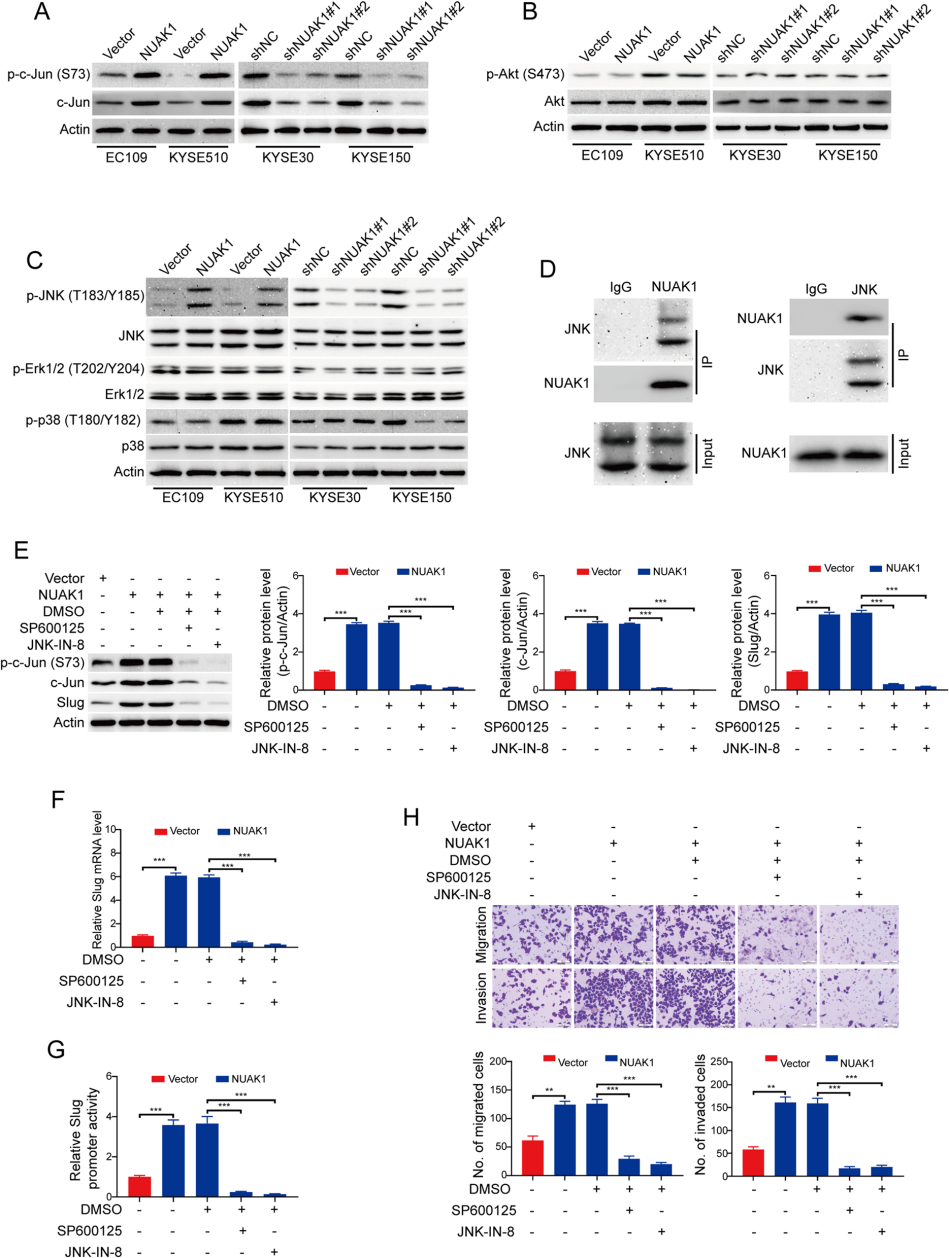
NUAK1 activates the JNK/c-Jun pathway to derive Slug-mediated migration and invasion in ESCC cells. **A**–**C** Western blot analysis of p-c-Jun (S73) and c-Jun (**A**), p-Akt (S473) and Akt (**B**), p-JNK (T183/Y185), JNK, p-Erk (T202/Y204), Erk, p-p38 (T180/Y182) and p38 (**C**) in NUAK1-overexpressing EC109 and KYSE510 cells, NUAK1-knockdown KYSE30 and KYSE150 cells and control cells. **D** KYSE30 cell lysates were subjected to immunoprecipitation with IgG, anti-NUAK1 (left panel) or anti-JNK (right panel) antibodies. Immunoprecipitates were blotted with indicated antibodies. **E** The expression of p-c-Jun (S73), c-Jun and Slug was analyzed in NUAK1-overexpressing EC109 cells treated with SP600125 and JNK-IN-8. Quantification of indicated protein levels of n = 3 independent biological experiments (right panel). **G** The promoter activity of Slug was analyzed using the luciferase assay in NUAK1-overexpressing EC109 cells treated with SP600125 and JNK-IN-8. H, Cell migration and invasion were analyzed in NUAK1-overexpressing EC109 cells treated with SP600125 and JNK-IN-8. Error bars denote mean ± SD. **P < 0.01; ***P < 0.001. *P* value was calculated by one-way ANOVA with post hoc intergroup comparison by the Tukey’s test (for **F**–**H**)

It has been reported that phosphorylation of c-Jun at Ser73 protects c-Jun from ubiquitination and subsequent degradation to increase its transcriptional activity [26]. We, therefore, examined whether NUAK1 influences the phosphorylation of c-Jun using Western blotting. As expected, forced overexpression of NUAK1 attenuated the protein levels of c-Jun and p-c-Jun (Ser73) (Fig. 6A and Additional file 1: Fig. S2A). Conversely, the protein levels of total c-Jun and its phosphorylation at Ser73 was diminished in NUAK1-silenced ESCC cells (Fig. 6A and Additional file 1: Fig. S2A). Next, we sought to explore the molecular mechanism by which NUAK1 regulates the phosphorylation of c-Jun. Overwhelming evidence showed that Akt [7] and mitogen-activated protein kinases (MAPKs) [27–29] are all able to phosphorylate c-Jun at Ser73 sites, we therefore examined whether NUAK1 regulates the activity of these proteins. Western blot analysis showed that the protein levels of p-Akt (S473) and total Akt were not obviously changed by NUAK1 (Fig. 6B and Additional file 1: Fig. S2B). Next, we investigated whether the family members of MAPKs such as JNK, ERK and p38 are involved in the activation of c-Jun through phosphorylation. Western blot analysis showed that the protein level of p-JNK (T183/Y185) was obviously enhanced in NUAK1-overexpressing ESCC cells, whereas was abrogated in NUAK1-depleted KYSE30 and KYSE150 cells, compared with their control groups, respectively (Fig. 6C and Additional file 1: Fig. S2C). However, overexpression or knockdown of NUAK1 has no considerable change in the levels of JNK, p-Erk (T202/Y204), Erk and p38 (Fig. 6C and Additional file 1: Fig. S2C). The expression of p-p38 (T180/Y182) was upregulated in KYSE30 cells, but was downregulated in KYSE150 cells (Fig. 6C and Additional file 1: Fig. S2C). Furthermore, Co-IP assay also validated the interaction between NUAK1 and JNK (Fig. 6D). To further determine the role of JNK in NAUK1-mediated upregulation of c-Jun in ESCC cells, NUAK1-overexpressing EC109 cells were treated with two specific JNK inhibitors SP600125 and JNK-IN-8 for 24 h, and then the cell lysates were collected and subjected to Western blot analysis. As illustrated in Fig. 6E, pharmacological inactivation of JNK1/2 kinase by SP600125 and JNK-IN-8 remarkably diminished the expression of p-c-Jun (Ser73) and c-Jun. As expected, the protein and mRNA levels of Slug were also concomitantly inhibited (Fig. 6E, F). Consistently, luciferase reporter assay showed that the promoter luciferase activity of Slug in NUAK1-overexpressing cells was obviously impaired by the two tested inhibitors of JNK (Fig. 6G). More importantly, pharmacological inhibition of JNK1/2 also significantly attenuated the migration and invasion of NUAK1-overexpressing EC109 cells detected by transwell migration and invasion assay (Fig. 6H).

### NUAK1 promotes tumor metastasis in nude mice

**Fig. 7:**
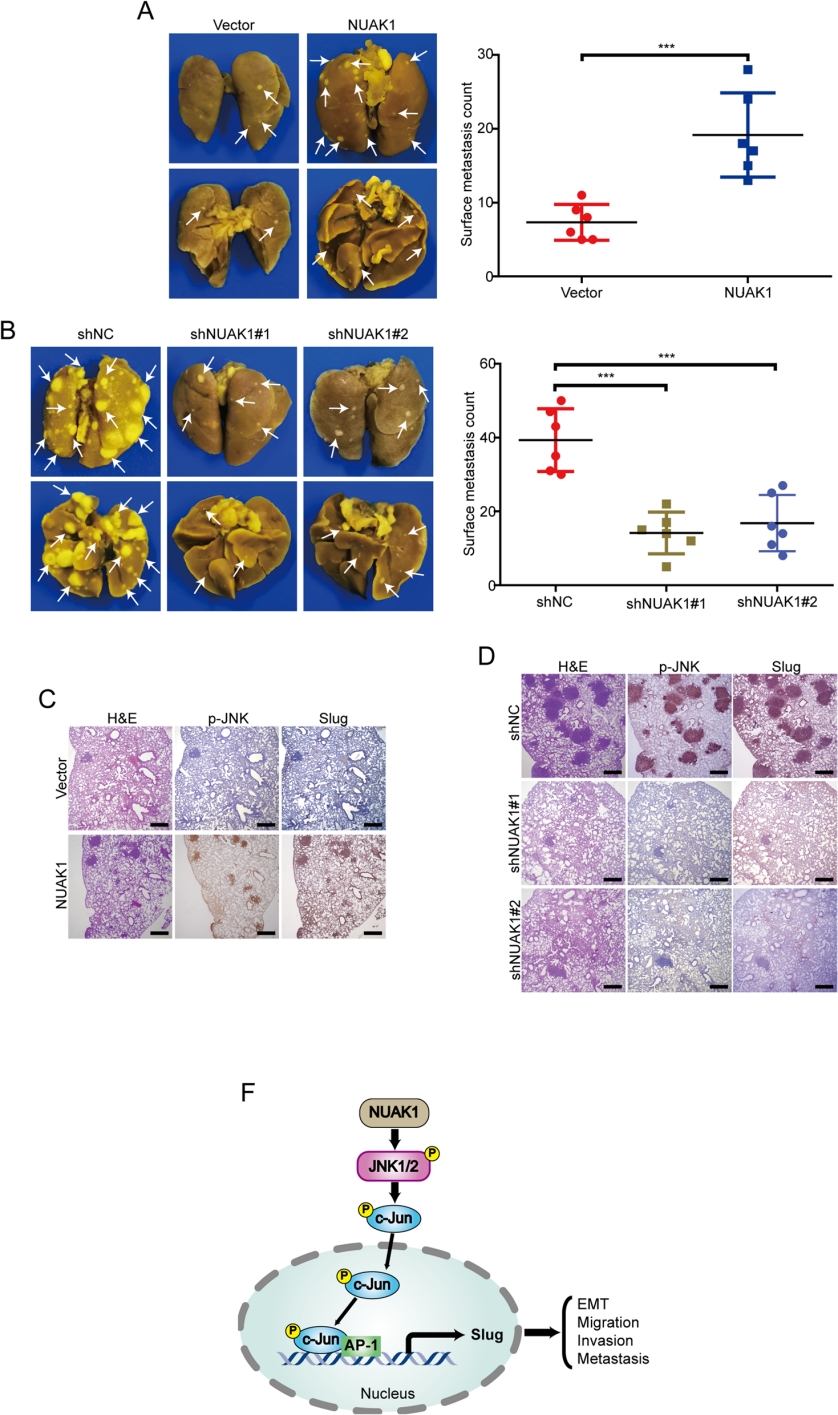
NUAK1 promotes ESCC cell metastasis in nude mice.** A** Representative images of lung nodules formed by NUAK1-overexpressing EC109 cells and control vector cells are shown. The numbers of lung metastatic nodules are shown in the right panel. *P* value was calculated by Student’s t-test. **B** Left, representative images of lung tissues from animals bearing xenografts from KYSE30 cells with stable NUAK1 knockdown. Right, the dot plots show the quantitative analysis of metastatic nodules on the lung surface in each group. *P* value was calculated by one-way ANOVA with post hoc intergroup comparison by the Tukey’s test. **C** The expression of p-JNK (T183/Y185) and Slug was evaluated by IHC in lung tissues from EC109-NUAK1 and vector cells. **D** Representative immunostaining of p-JNK (T183/Y185) and Slug in lung tissues from KYSE30-NUAK1 shRNA and control shNC cells. Scale bars, 500 μm. Error bars denote mean ± SD. ***P < 0.001. E, The working model of NUAK1 in ESCC

Considering that ectopic expression of NAUAK1 significantly enhanced the migration and invasion of ESCC cells in vitro, we then determined to further analyze the effect of NUAK1 overexpression on tumor metastasis in vivo. 1 × 10^6^ EC109 cells were injected into BALB/c-nu/nu mice via the tail vein to establish a metastatic xenograft model and the tumor metastasis were examined after 8 weeks. As shown in Fig. 7A, mice injected with NUAK1-EC109 cells formed more nodules on the lung surface than those injected with Vector- EC109 cells. To further confirm this conclusion, we injected KYSE30-shNUAK1 cells (1 × 10^6^) into the nude mice via lateral tail vein and then examined the tumor metastasis after 8 weeks. The results showed that the number and size of metastatic nodules in the lungs was much smaller in NUAK1-knockdown group than that of the control group (Fig. 7B). More importantly, IHC staining also showed that NUAK1 promoted the levels of p-JNK (T183/Y185) and Slug in lung metastasis lesions derived form NUAK1-overexpressing cells, compared with that of the control group (Fig. 7C). Vice versa, the expression of p-JNK (T183/Y185) and Slug in lung metastasis lesions was much lower in NUAK1-knockdown cells that those of cells transfected with shNC (Fig. 7D). Taken together, these data indicate that NUAK1 promotes ESCC metastasis through JNK/c-Jun/Slug signaling pathway (Fig. 7E).

## Discussion

In this study, our results showed that NUAK1 was highly expressed in ESCC tissues but weakly expressed in paired normal esophageal epithelial specimens. Additionally, high NUAK1 expression was positively associated with tumor tissue invasion, lymph node metastasis, poor pathological TNM stage and poor outcomes of patients with ESCC. These findings suggest that NUAK1 is a potential diagnostic biomarker for ESCC patients. Loss- and gain-of-function studies demonstrated that expression of NUAK1-targeting shRNA substantially suppressed ESCC cell EMT, migration and invasion in vitro and abrogated the metastatic behavior of ESCC cancer cells in xenograft models, indicating targeting NUAK1 is a promising strategy for the treatment of ESCC patients.

As a serine/threonine kinase, NUAK1 has been shown to play an oncogene in various human cancers. In colorectal cancer, depletion or inhibition of NUAK1 renders human colorectal cancer cells and murine colorectal tumors vulnerable to oxidative stress-induced cell death [12]. Loss of NUAK1 has recently been showed to trigger genomic instability and suppress tumor cell growth in pancreatic cancer [11]. Moreover, in epithelial ovarian cancer, NUAK1 promotes cell adhesion, spheroid compaction and metastasis through regulation of fibronectin expression and matrix production [20]. Relevant to our own findings, an elevated NUAK1 expression is positively associated with poor prognosis in ESCC patients. Interestingly, using gain-and loss-of function studies, we demonstrated that NUAK1 hardly influences ESCC cell growth and colony formation, but greatly promotes EMT, migration, invasion and metastasis. Our findings were in agreement with a previous report, which showed that knockdown of NUAK1 expression remarkably suppresses gastric cancer cell invasion and metastasis via regulating EMT, rather than proliferation in vitro and in vivo [18]. Altogether, those results from us and others suggest that targeting NUAK1 is a promising strategy to treat metastatic malignancies.

EMT is a dynamic cellular process in which cells lose cell-cell junctions and baso-apical polarity and acquire mesenchymal features with increased motility and invasive potential [5]. In cancer, EMT has been associated with various tumor functions, including tumor initiation, malignant progression, migration, invasion and metastasis [5, 6]. As a family member of the snail, Slug has been described as a suppressive transcriptional factor of the epithelial marker E-cadherin and inducer of EMT and invasion [30–32]. Increased expression of Slug has been observed in in several human cancers including ESCC [33], breast cancer [31], hepatocellular carcinoma [34] and gastric cancer [18]. In ESCC, the high Slug expression was associated with tumor tissue invasion, poor pathological stage and reduced disease free survival, and may serve as a diagnostic biomarker and as a predictor of poor disease prognosis for ESCC patients [33, 35]. Although several studies previously showed that high expression of NUAK1 is positively associated with EMT, migration and invasion in gastric cancer [18], ovarian cancer [36], pancreatic cancer [37] and lung cancer [13], the underlying molecular mechanisms remain poorly understood. In this study, we demonstrated that NUAK1 promoted ESCC cell EMT, migration and invasion at least in part through activating the transcription of Slug, which was based on the following facts: (1) Enforced expression of NUAK1 drastically promoted the expression of Slug at both mRNA and protein levels. (2) In NUAK1-over expressing cells, the knockdown of Slug abrogated tumor cell EMT, migration and invasion induced by NUAK1. (3) Ectopic expression of Slug rescued the inhibitory effects of NUAK1 shRNA on ESCC cell EMT, migration and invasion. Consistent with our findings in this study, a previous report has showed that knocking down NUAK1 remarkably suppressed invasion and metastasis, as well as down-regulation of the mTOR/p70S6k signals, Slug and SIP1 in gastric cancer [18]. Together, these findings confirmed that EMT-related transcription factor Slug is critical for NUAK1-mediated metastasis.

Emerging evidence suggests that JNK, a family member of mitogen-activated protein kinase (MAPK) signaling, regulates many physiological processes, including inflammatory responses, differentiation, cell proliferation, morphogenesis, survival and death [38]. In addition, it is increasingly apparent that JNK is also a key contributor to the cancer development and progression and plays a crucial role in several aspects of tumorigenesis, including cell differentiation, proliferation, invasion, angiogenesis, apoptosis, and metastasis [39]. In ESCC, the activation of the JNK signaling pathway has been described as a mediator of RAD18 to induce ESCC cell migration and invasion [40]. Consistent with these findings, in this study, we demonstrated a direct protein-protein interaction between NUAK1 and JNK using Co-IP assay. We further found that NUAK1 enhanced the phosphorylation of JNK and subsequently increased the transcription of Slug, whereas inhibiting JNK using two specific inhibitors, SP600125 and JNK-IN-8, remarkably abrogated NUAK1-induced expression of Slug. In addition, both SP600125 and JNK-IN-8 were able to diminish the migration and invasion induced by NUAK1 in tested ESCC cells. JNK was previously reported to induce c-Jun phosphorylation to enhance the binding of c-Jun to the promoters of oncogenes, thereby increasing its transcriptional activity [41]. Consistently, in this study, we found that treatment with JNK inhibitors in NUAK1-overexpressing cells dramatically decreased the promoter activity of Slug induced by NUAK1. Taken altogether, we confirmed that the highly elevated NUAK1 promoted the migration, invasion and metastasis of ESCC cells by activating the JNK-Slug signaling pathway.

## Conclusions

In summary, our findings demonstrated that NUAK1 promote ESCC cell migration, invasion and metastasis at least partly through activation of JNK/c-Jun/Slug signaling pathway. In addition, as depletion of NUAK1 greatly inhibited the migration and invasion in vitro and metastasis in vivo, indicating targeting NUAK1 is a promising therapeutic strategy for metastatic ESCC.

### Supplementary Information

**Additional file 1: Figure S1.** NUAK1 promotes EMT via upregulation of Slug. **Figure S2.** NUAK1 activates the JNK/c-Jun pathway in ESCC cells. **Table S1.** Primers for qRT-PCR analysis. **Table S2.** Correlation between NUAK1 expression and clinicopathological parameters in 116 cases of ESCC.

## Data Availability

All data generated or analyzed during this study are included in this published article and its Additional files.
