# Supplementary material for: NUAK1 promotes tumor metastasis through upregulating slug transcription in esophageal squamous cell carcinoma
Source: Cancer Cell Int. 2023 Nov 2;23:258. doi: 10.1186/s12935-023-03101-7 (PMC10621130; doi:10.1186/s12935-023-03101-7)
Supplement: Supplementary file 1 — Additional file 1: Figure S1. NUAK1 promotes EMT via upregulation of Slug. Figure S2. NUAK1 activates the JNK/c-Jun pathway in ESCC cells. Table S1. Primers for qRT-PCR analysis. Table S2. Correlation between NUAK1 expression and clinicopathological parameters in 116 cases of ESCC. [file 12935_2023_3101_MOESM1_ESM.docx]

**
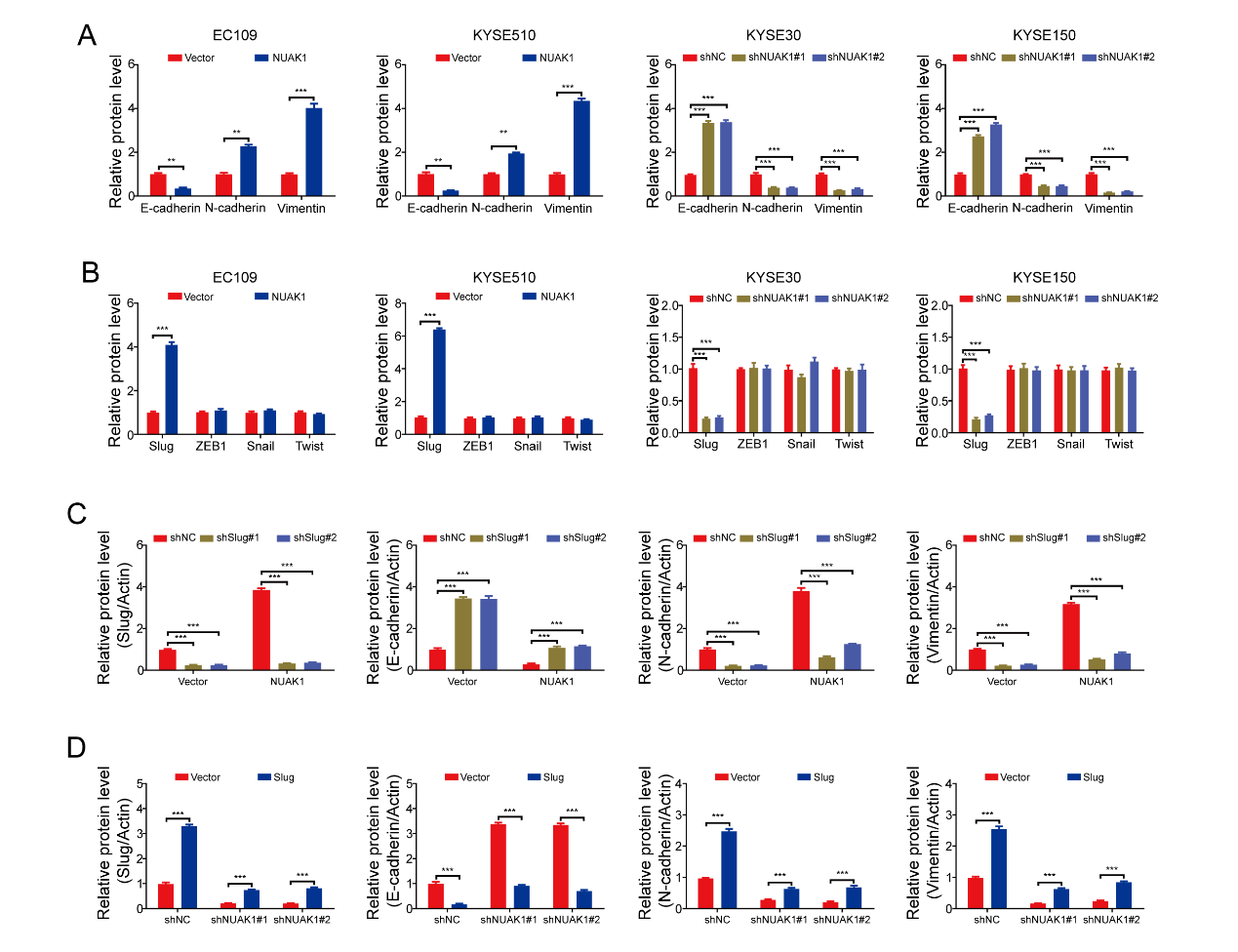
**

**Fig. S1 NUAK1 promotes EMT via upregulation of Slug.** A, Quantification of the protein levels of E-cadherin, N-cadherin and Vimentin in NUAK1-overexpressing (EC109 and KYSE510) or knockdown (KYSE30 and KYSE150) ESCC cells. B, Quantification of the protein levels of Slug, ZEB1, Snail and Twist in NUAK1-overexpressing (EC109 and KYSE510) or knockdown (KYSE30 and KYSE150) ESCC cells. C, Quantification of the protein levels of Slug, E-cadherin, N-cadherin and Vimentin in NUAK1-overexpressing EC109 cells treated with Slug shRNA. D, Quantification of the protein levels of Slug, E-cadherin, N-cadherin and Vimentin in NUAK1-knockdown KYSE30 cells treated with Slug and control plasmids. *P* value was calculated by Student's t-test or one-way ANOVA with post hoc intergroup comparison by the Tukey’s test. Error bars denote mean ± SD. Data are representative of three independent experiments. **P* < 0.05, ***P* < 0.01, ****P* < 0.001.

**
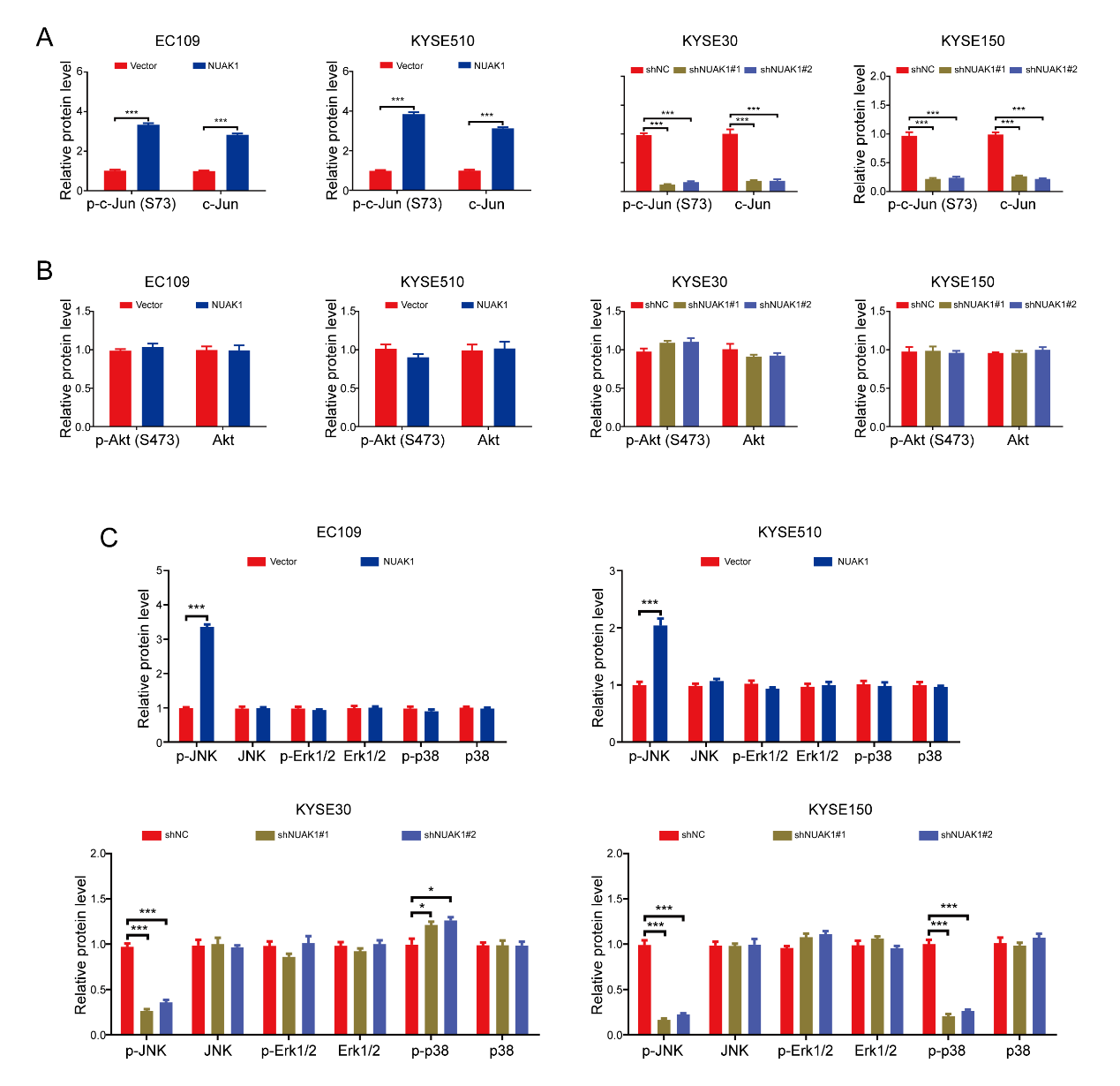
**

**Fig. S2 NUAK1 activates the JNK/c-Jun pathway in ESCC cells.** A, Quantification of the protein levels of p-c-Jun (S73) and total Jun in NUAK1-overexpressing (EC109 and KYSE510) or knockdown (KYSE30 and KYSE150) ESCC cells. B, Quantification of the protein levels of p-Akt (S473) and total Akt in NUAK1-overexpressing (EC109 and KYSE510) or knockdown (KYSE30 and KYSE150) ESCC cells. C, Quantification of the protein levels of p-JNK, JNK, p-Erk1/2, Erk1/2, p-p38 and p38 in NUAK1-overexpressing (EC109 and KYSE510) or knockdown (KYSE30 and KYSE150) ESCC cells. *P* value was calculated by Student's t-test or one-way ANOVA with post hoc intergroup comparison by the Tukey’s test. Error bars denote mean ± SD. Data are representative of three independent experiments. **P* < 0.05; ****P* < 0.001.

**Table S1. Primers for qRT-PCR analysis.**

| Genes | Sense primer | Antisense primer |
| --- | --- | --- |
| NUAK1 | 5'-GCAAAGGGGAGCTGTACGAT-3' | 5'-TGGAAAGCCCAAAGTCAGCA-3' |
| Slug | 5'-CTGGGCGCCCTGAACATGCAT-3' | 5'-GCTTCTCCCCCGTGTGAGTTCTA-3' |
| GAPDH | 5'-ATTCCACCCATGGCAAATTCC-3' | 5'-GACTCCACGACGTACTCAGC-3' |

**Table S2. Correlation between NUAK1 expression and clinicopathological parameters in 116 cases of ESCC.**

| Variable | Low NUAK1 | High NUAK1 | *P* value |
| --- | --- | --- | --- |
| Age |  |  | 0.448 |
| ≤60 | 21 | 25 |  |
| >60 | 37 | 33 |  |
| Gender |  |  | 0.399 |
| Male | 38 | 30 |  |
| Female | 20 | 22 |  |
| Differentiation |  |  | 0.468 |
| Well | 5 | 3 |  |
| Moderate | 32 | 28 |  |
| Poor | 21 | 27 |  |
| Tumor location |  |  | 0.120 |
| Upper | 3 | 4 |  |
| Middle | 33 | 35 |  |
| Lower | 12 | 19 |  |
| Tumor invasion depth (T) |  |  | **0.003** |
| T1-2 | 33 | 17 |  |
| T3-4 | 25 | 41 |  |
| Lymph node metastasis (N) |  |  | **0.009** |
| N0 | 34 | 20 |  |
| N1-3 | 24 | 38 |  |
| Pathological TNM stage |  |  | **0.022** |
| I/II | 28 | 16 |  |
| III/IV | 30 | 42 |  |

*P* value calculated by the Pearson Chi-square test or Fisher’s exact test; Values in bold signify *P* < 0.05, which is considered significant.
